# Sensorineural Hearing Loss in Patients With Chronic Kidney Disease: A Comprehensive Review

**DOI:** 10.7759/cureus.48244

**Published:** 2023-11-03

**Authors:** Manasi Agrawal, Chandra Veer Singh

**Affiliations:** 1 Pathology, Jawaharlal Nehru Medical College, Datta Meghe Institute of Higher Education and Research, Wardha, IND; 2 Otolaryngology - Head and Neck Surgery, Jawaharlal Nehru Medical College, Datta Meghe Institute of Higher Education and Research, Wardha, IND

**Keywords:** labyrinth, hemodialysis, end-stage renal failure, sensorineural (sn) hearing loss, chronic kidney disease (ckd)

## Abstract

This article aims to ascertain the prevalence of loss of hearing in patients with chronic kidney disease (CKD) and also to examine potential causes of sensorineural hearing loss (SNHL) in patients suffering from CKD. It has been discovered in recent years that there is a relationship between the occurrence of SNHL and CKD. Nowadays many people are suffering from CKD. These patients deal with several otorhinolaryngological issues, such as SNHL, candidiasis, epistaxis, halitosis, dysgeusia, xerostomia, and lip and thyroid malignancies. One of the most frequent otorhinolaryngological complications is audiovestibular system impairment. There are various proposed mechanisms for the appearance of loss of hearing in people suffering from CKD. The kidney and the inner ear have multiple functional and structural similarities, which may be the cause of these problems in CKD patients. In addition, changes in the homeostasis of water and electrolytes can affect the endolymphatic fluid and result in endolymphatic hydrops. Finally, some medications, like aminoglycosides and loop diuretics, are well known for their ototoxicity and are utilized to treat patients with CKD. Only a small number of population-based research have so far been able to show a connection between CKD and audiovestibular system impairment. Some investigation has shown that CKD patients are more likely than healthy people to experience vestibular impairment.

The quality of life of a patient can be reduced by hearing loss. People with hearing loss experience communication issues in daily life, which negatively affects their cognitive and psychosocial functioning. Social isolation and a poor quality of life in terms of health can all result from hearing loss. In addition, decreased renal function has also been linked to poor quality of life, hospitalization, and cognitive dysfunction.

## Introduction and background

The most crucial hearing organ is the ear. It has three sections: 1) external ear, 2) middle ear, and 3) inner ear. The term "labyrinth" also refers to the inner ear. It comprises the cochlea, which is important for hearing, and the vestibular apparatus, which controls balance. Sound energy from the environment passes through the external and middle ear and is transformed into mechanical energy. The labyrinth then receives this mechanical energy. Then the labyrinth converts this mechanical energy into electrical energy. The final organ for hearing is the cochlea. The functional hearing processing unit is present inside the cochlea and is called the organ of Corti. This organ has an extreme sensitivity to the surrounding chemicals. Ototoxicity, or toxic damage to the ear, is caused by changes in the organ of Corti's physiological environment. Several factors deteriorate the labyrinth's functionality. These include aging, drugs, and some acquired and congenital disorders [1].

The kidney is the organ that is most crucial for the removal of toxic chemicals from the body, thus it provides an ideal environment for the vital organs to work properly. Different renal disorders harm the labyrinth, it is because toxic compounds build up in the blood which impairs the inner ear’s functions. The nephron is a functioning unit of the kidney and it resembles the stria vascularis found in the inner ear both structurally and functionally. Due to this link, both organs are susceptible to the same agents and genetic alterations while still in the womb [2].

**Figure 1:**
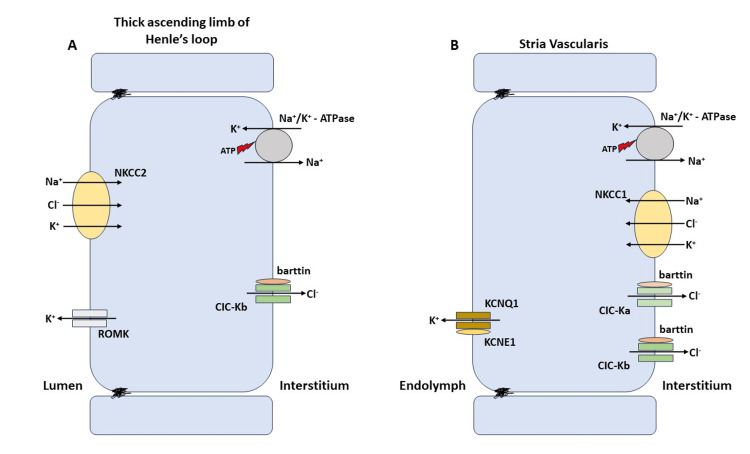
Ion channels and transporters present in both the inner ear and kidney Image Credit: Author Manasi Agrawal

The voltage-dependent movement of Cl^-^ ions through the cell membrane is mediated by the ClC proteins [3]. The CLC gene family controls these proteins. They form Cl^-^/H^+^ antiporters, Cl^-^ channels, and CLC-K channels. The essential subunit of the ClC-K channel is the barttin protein. Both the kidney and the labyrinth contain these transporters and channels [3-5]. The nephron's luminal NKCC2 transporters cause the collection of sodium ions, potassium ions, and chloride ions inside the cells. Potassium ion moves back into the lumen by ROMK1 channels, while Cl^-^ and Na^+^ are absorbed again into the circulation via ClC-kb channels and Na^+^/K^+^ ATPase, respectively. Basolateral NKCC1 transporters in the stria vascularis aid in the movement of K^+^, Na^+^, and Cl^-^ within the cells. ClC-Ks isomers and Na^+^/K^+^ ATPase, respectively, aid in the extrusion of Cl^-^ and Na^+^ back into the interstitium. K^+^ levels rise in the endolymph due to the activity of the KCNQ1/KCNE1 channel, which is necessary for the sensory transduction of inner hair cells [3]. Both the basolateral membranes of the thick and thin ascending limbs of the Henle’s loop as well as the marginal cells of the labyrinth's stria vascularis contain CLC-K/Barttin [4]. They are essential for the reabsorption of NaCl in the kidneys and for the formation of endolymph in the labyrinth (Figure 1 shows ion channels and transporters present in both the inner ear and kidney).

## Review

Loss of hearing in chronic kidney diseases

The quality of life of a patient can be reduced by hearing loss [6]. Chronic kidney disease (CKD) is a world health problem. Compared to the general population, people suffering from CKD have a higher incidence of sensorineural hearing loss (SNHL), which ranges from mild hearing loss in 76% of cases [7], to medium severe loss of hearing in 47% of individuals who underwent testing [2]. Variable nations have quite variable rates of loss of hearing among CKD patients. It was reported to be 64% in India [8], 68% in Nigeria [9], 45% in Iran [10], and 63% in Croatia [11]. A higher prevalence of hearing loss (between 71% to 76%) was seen in earlier investigations [12,13]. This difference might result from variations in patient ages, hearing loss testing techniques, or the duration of CKD and hemodialysis treatment [14]. Most of the loss of hearing is in the high-frequency range with a notch at 6 kHz. The type of hearing loss can distinguish between lesions in the inner ear and the neural pathways (SNHL) or lesions in the external and tympanic cavity (conductive type of loss of hearing), while the severity of loss of hearing can demonstrate the extent of impairment to the auditory system.

In people suffering from kidney failure, uremia affects almost every organ system of our body [15]. Numerous studies have shown that individuals suffering from CKD are more likely to develop otologic symptoms linked to the dysfunction of the audiovestibular system. These symptoms are typically persistent and challenging to manage. The possible cause connecting chronic kidney disease with SNHL is still uncertain. Nevertheless, the stria vascularis and the nephrons exhibit notable physiological, anatomical, and pharmacological similarities [16]. In the stria vascularis, immunoglobulins made against nephrons are also deposited [17,18]. Additionally, the labyrinth may experience electrolytic and osmotic alterations associated with CKD that also impact the cochlea [19]. Further, electrolytic disturbances and osmotic changes can also be induced by hemodialysis and kidney transplantation which results in SNHL, vertigo, and tinnitus. Also, patients with CKD may experience hearing loss due to deficiency of vitamin D, hypertension, and high serum urea levels [9,20,21]. Hearing characteristics can alter due to abnormalities in the cationic gradient of endolymphatic fluid [22]. Additionally, “uremic neuropathy,” changes in the peripheral nervous system (PNS) and central nervous system (CNS), can lead to hearing loss linked to CKD [23,24].

Causes of loss of hearing in patients with CKD

**Figure 2:**
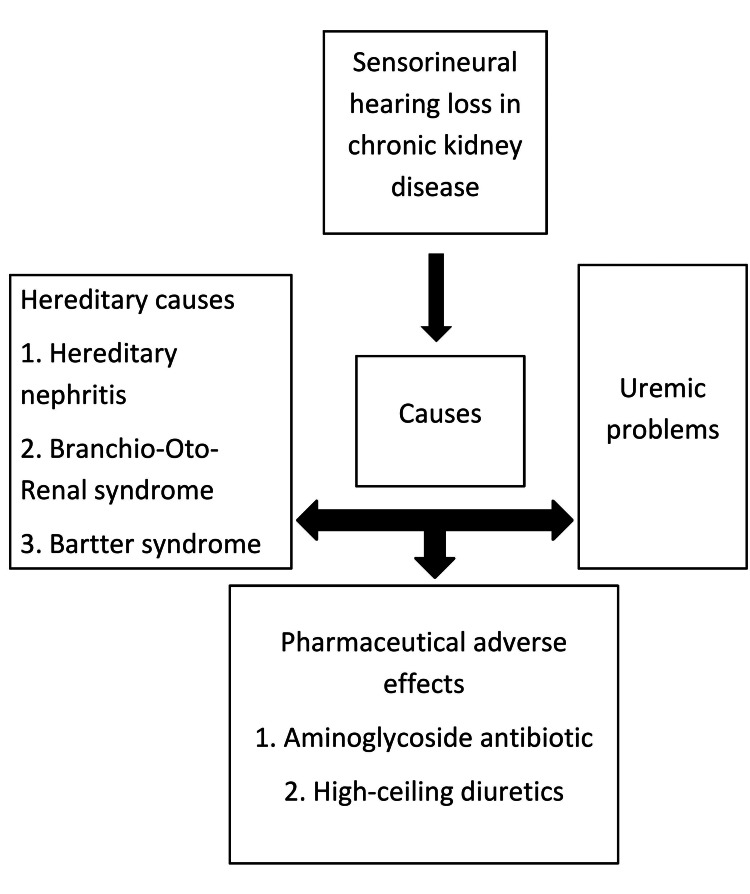
Causes of loss of hearing in patients with chronic kidney disease Image Credit: Author Manasi Agrawal

People suffering from CKD may experience hearing loss due to a variety of reasons viz, heredity, uremic problems, and pharmaceutical adverse effects (Figure 2 shows causes of loss of hearing in CKD patients) [2,22,25].

Genetic Causes

Hereditary nephritis: Alport syndrome (AS) is commonly known as hereditary nephritis. It is a well-known causative factor of CKD in young people. Gradual renal failure, distinctive ocular abnormalities, and SNHL are hallmarks of AS [25]. One in 5000 people is said to be affected by AS, which accounts for 0.6% of newly diagnosed cases of end-stage renal disease (ESRD) in adults and 13% of cases in children [26,27]. Type IV collagen makes up the glomerular basement membrane (GBM). Type IV collagen has six distinct α chains, numbered from α1 to α6, which together form triple helix structures in which 3 α chains are united. The triplet of α3- α4- α5 is present in the GBM, the basement of the cochlea, and the base of the eye lens, whereas α5- α5- α6 is present in the malpighian capsule and the basement membrane of the epidermis. When malformation takes place in the α-chain, these triple helix structures are destroyed resulting in a decline in kidney function, lesions in the eyes, and SNHL.

**Figure 3:**
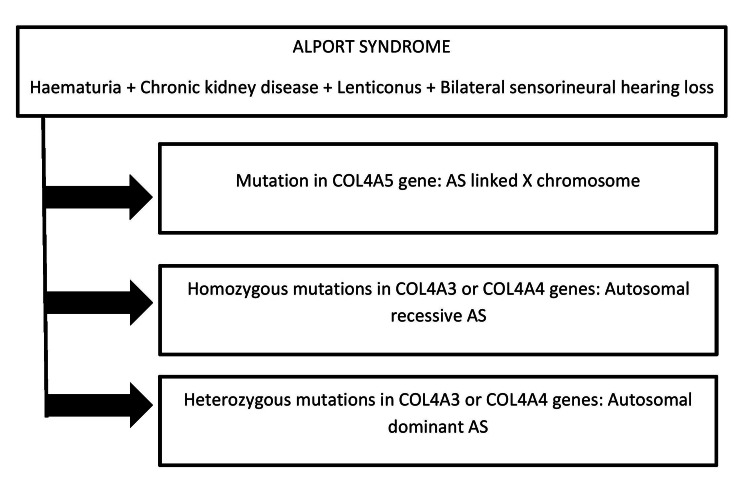
Classification of the Alport syndrome Image Credit: Author Manasi Agrawal

The type IV collagen α3, α4, and α5 chains are coded by the genes COL4A3, COL4A4, and COL4A5, respectively. The alterations in the above-mentioned genes result in AS [28]. Depending on how it is inherited, AS is classified as X-linked AS (XLAS), autosomal dominant AS (ADAS), and autosomal recessive AS (ARAS) (Figure 3 shows the classification of Alport syndrome).

AS occurs due to the mutation in the gene encoding for type IV collagen [25]. Due to this genetic abnormality, the basement membrane becomes thick and often split, resulting in the recognizable “basket weave” pattern. Alterations in the COL4A5 gene give rise to AS linked to the X chromosome in almost 86% of the patients. This gene is present on chromosome Xq22 coding the α5-chain of type IV collagen. About 14% of cases have autosomal recessive AS, and autosomal-dominant inheritance has been noted in a few cases without platelet abnormalities and some cases with simultaneous thrombocytopathy [25]. Initial symptoms of the illness include asymptomatic microscopic haematuria, which occasionally coexists with episodes of massive haematuria. Though the progression rate is quite variable, worsening proteinuria and ESRD may develop [25]. People with AS frequently exhibit bilateral high-frequency SNHL [25]. Nevertheless, some infected people with the X-linked nephritis that progresses to ESRD may not have a loss of hearing, which could result in a missed diagnosis. Because the stria vascularis of the labyrinth and the GBM share a connective tissue structure (type IV collagen), many cases of AS affect both organs [25,29].

Branchio-oto-renal (BOR) syndrome: Ear and kidney problems occur simultaneously in BOR syndrome. Bilateral hypoplasia or dysplasia, bilateral renal agenesis, unilateral renal agenesis with contralateral hypoplasia or dysplasia, vesicoureteric reflux, and ureteropelvic obstruction are all examples of renal abnormalities. The glomerular filtration rate of the kidneys might be normal or severely reduced. Ear abnormalities include preauricular pits, hearing loss, and deformity in the external ear, middle ear, and labyrinth [25].

Branchiogenic malformations (branchial fistula or cyst), loss of hearing, and kidney problems, including congenital deformities of the kidney and urinary tract, are features of the autosomal dominant condition known as BOR syndrome [30]. About 1 in 40,000 people in the overall population and 2% of children who are profoundly deaf have BOR syndrome [31]. The term branchiootic syndrome (BOS) can also be used if there isn’t a kidney abnormality. Doctors find it challenging to diagnose BOR syndrome because of the high clinical variability. BOR syndrome is an uncommon hereditary disorder that has an impact on many organ systems. BOR syndrome is extremely rare, however, numerous cases have been documented since it was initially identified. In 2004, Abdelhak et al. [32] suggested the major and minor criteria which are the commonly accepted criteria for diagnosis of BOR syndrome. Loss of hearing, preauricular pits, renal abnormalities, and second branchial anomaly were the major criteria. Preauricular tags, asymmetry of the face, middle ear abnormalities, inner ear abnormalities, external ear abnormalities, and palate abnormalities were the minor criteria. BOR syndrome is diagnosed in patients without a family history if they satisfy three or more of the major criteria listed above, or two major criteria and at least two minor criteria. According to Stinckens et al. [33], the incidences of primary characteristics of BOR syndrome were 96% hearing impairment, 87% second branchial arch fistula/cyst, 87.2% malformed auricles, 57% renal abnormalities, and 86% preauricular sinus. Most patients do not exhibit any symptoms of renal disease in the early stages [34].

Bartter syndrome type IV: The thick ascending limb of Henle’s loop contains the majority of the ClC-Kb/Barttin protein, which is primarily used to reabsorb salt and then water [35]. In this area of the nephron, the basolateral Na^+^/K^+^ pump produces a Na+ electrochemical gradient which triggers the secondary active transport of NKCC2, which is expressed at the apical membrane, which causes sodium ion, chloride ion, and potassium ion to accumulate inside the cell. Potassium ions are returned to the lumen by ROMK K^+^ channels (which are also expressed at the apical membrane), whereas Cl^-^ and Na^+^ are reabsorbed by the interstitial fluid through the ClC-Kb channels and Na^+^/K^+^ pump respectively. In the basolateral membrane of marginal cells of the stria vascularis of the labyrinth, both ClC-K isomers are expressed. The endolymph of the scala media has a higher content of potassium ions and a higher positive potential (about a hundred mV more than ordinary extracellular fluids) because of this multilayered epithelium. Both of which are crucial for hearing. In marginal cells of the stria vascularis Na^+^/K^+^ pumps and NKCC1 transporters build up potassium ions and chloride ions inside the cells. While apical KCNQ1/KCNE1 K+ channels leak excessive K^+^ into the endolymph and ClC-K/Barttin channels return chloride ions into the interstitial fluid [36].

Salt-losing Bartter syndrome type III is caused by a mutation in the gene encoding ClC-Kb [37]. This syndrome is marked by metabolic alkalosis, hypokalemia, and secondary high aldosterone level with low or normal BP [38]. Because both ClC-K proteins are inactive in the unavailability of barttin, alterations in the gene encoding barttin result in Bartter syndrome type IV, which combines salt wasting and congenital deafness. When only one ClC-K channel is impaired, as in the case of ClC-Kb alterations in Bartter type III, hearing is unaffected since the other isomer channel however provides the necessary Cl^-^ recycling. Disruption of either the Barttin or both ClC-K channels results in deafness [39,40].

Adverse effects of medicines

More than 451 medications are known to be harmful to the ear. These include both over-the-counter medications like NSAIDs (pain relievers) and prescription medications like cancer medications, antibiotics, antimalarials, and diuretics. In the majority of cases, this type of toxicity to the ear is often an acute, short-lived adverse effect; if the individual stops the utilization of medicine, the symptoms usually go away. This is not the case, although, aminoglycoside and derivatives of platinum are utilized for the treatment of cancer which can result in irreversible loss of hearing [41]. The mechanism of ototoxicity caused by medicines is different.

High-Ceiling Diuretics and Aminoglycoside Antibiotics

For patients suffering from chronic kidney disease, the medicines of importance in causing toxicity to the ear are the aminoglycoside antibiotics, which are frequently utilized for the control of septicemia and urinary tract infection (UTI) which are commonly seen in such individuals, and furosemide, a loop diuretic, is also utilized for management of pulmonary edema and fluid overload in the patients suffering from CKD. However, many medical professionals are aware of the ototoxic effects of aminoglycosides, lack of diagnostic resources in many hospitals around the globe prevents a thorough examination of these potential adverse effects. Many regions lack effective treatment interventions for hearing loss, which has diminished interest in testing for this adverse effect. The toxicity caused by furosemide to the ear is frequently ignored by nurses and other medical personnel. When the medication is given more quickly and as an i.v. push medication, furosemide toxicity usually occurs.

Hearing loss among people receiving hemodialysis

Another category of CKD patients that experience hearing loss are those who are on hemodialysis and are in the end stage of the disease. Many articles show that people suffering from CKD have higher incidences of SNHL than the general population. It ranges from 26% to 78% [12,42]. However, all frequencies can be disturbed by CKD but a loss of hearing at high frequencies is commonly seen [43]. Along with antigenic similarities [29], the kidney and cochlea also share similar physiological mechanisms, such as the active transport of water and salt accomplished by the glomeruli in the kidney and stria vascularis in the cochlea [2]. The hormonal, hydroelectrolytic, and systemic metabolic alterations linked to CKD have been proven to have an impact on the cochlea [44]. The severity and time period of the disorder, electrolyte abnormalities, ototoxic medications, age, co-occurring conditions like diabetes mellitus (DM) and hypertension (HTN), and hemodialysis are some of the components that may play a role in the etiopathogenetic mechanisms of hearing loss in chronic kidney disease [10,45,46].

In the study of Iraq, 59 patients were monitored for a year with a pure-tone audiometry (PTA) test every six months to investigate the impact of hemodialysis on the hearing threshold in adults with CKD [47]. The study started with 39 individuals (66.1%) who had SNHL. Six more patients experienced SNHL during the one-year follow-up, giving the study's final point prevalence rate of 75.8%. The loss of hearing was frequently observed at higher frequencies. About 64.4% of the individuals in the study had showed worsening hearing thresholds. The mean hearing threshold was 29.2 ± 21.1 dB at the beginning of the research and 36.9 ± 17.3 dB at the conclusion. Age, sex, blood urea, serum electrolytes, and the duration of CKD were not shown to be significantly associated with loss of hearing. The duration of hemodialysis was the only remarkable not dependent predictor of Sensorineural hearing loss, according to multivariate analysis [47].

Preventive healthcare strategies of SNHL

Due to the large number of persons undergoing long-term hemodialysis treatments worldwide and the increasingly widespread hearing loss among this group, which is typically ignored [12,42], it is appropriate to emphasize the need to develop awareness about preventative measures for the loss of hearing. Additionally, it has been determined that 122 million people globally have hearing loss (>42 dB, average 0.6-3 kHz) [48]. Genetic counseling for families known to possess abnormal genes should be the first preventive strategy. Through the use of improved antenatal care facilities, efforts should be made to address maternal issues such as low birth weight and premature deliveries during the prenatal period. Acute attention should be paid to perinatal/newborn asphyxia, neonatal jaundices requiring exchange transfusions, neonatal meningitis, etc. Increased vaccination campaigns should target measles, mumps, rubella, and meningitis in particular communities.

In clinical settings, ototoxic medications like the diuretic furosemide and the antibiotic aminoglycoside should be used with caution. When feasible, these drugs should not be given in combination. Education about the dangers of self-medication and the utilization of herbal products that might be toxic to the ear and kidneys, and also about issues such as undernourishment, overcrowding in nursery facilities, bottle feeding, inadequate housing systems, etc. is necessary. Acute respiratory infections should be better managed, noise levels should be reduced, and hearing protection should be used as necessary [48]. Comorbidities like diabetes mellitus and hypertension in adult patients should be identified and treated early. It is important to raise awareness among dialysis patients. Above all, rehabilitation programs for afflicted people should be put into place to enhance their quality of life through the use of implants and hearing aids.

## Conclusions

Otorhinolaryngological disorders are a common side effect of CKD and its therapy. CKD patients are more likely to experience SNHL, vestibular dysfunction, tinnitus, recurrent epistaxis, rhino-cerebral mucormycosis, oropharyngeal candidiasis, smell and taste changes, phonatory dysfunction, deep neck infections, mucosal abnormalities, halitosis, gingival hyperplasia, or xerostomia as opportunistic infections. Otorhinolaryngological dysfunctions associated with CKD are frequently chronic, challenging to manage, adversely affect the patient’s quality of life, and occasionally fatal. We concluded that CKD patients, particularly those following organ transplantation, require regular and ongoing examinations by an expert otorhinolaryngologist due to the high incidence of otorhinolaryngological problems caused by CKD itself and its treatment. It is especially important because some of these issues may be treatable if identified and treated early. Additionally, the introduction of appropriate treatment may be able to stop the advancement of some of these dysfunctions, and some dysfunctions may improve with therapeutic adjustment. Patients with CKD are predisposed to several otorhinolaryngological issues especially SNHL, and the link between SNHL and CKD has both been extensively explained, but the relationship between the remaining complications and CKD is still unknown.
